# 1638. Development of a Model to Estimate Persistence of hSBA Titers Over Time Following a Primary Series and Booster Dose of the Pentavalent Meningococcal MenABCWY Vaccine

**DOI:** 10.1093/ofid/ofad500.1472

**Published:** 2023-11-27

**Authors:** Bing Cai, Paula Peyrani, Johannes Beeslaar, Jason Maguire, Roger Maansson, Paul Palmer

**Affiliations:** Pfizer Inc., Eagleville, PA; Pfizer, Inc, Collegeville, Pennsylvania; Pfizer Vaccine Clinical Research and Development, Hurley, Berkshire UK, Hurley, Berkshire, England, United Kingdom; Pfizer Vaccine Clinical Research and Development, Pearl River NY, Pearl River, NY; Pfizer Vaccine Clinical Research and Development, Collegeville PA, Collegeville, PA; Pfizer Vaccine Medical Development, Scientific & Clinical Affairs , Collegeville PA, Collegeville, PA

## Abstract

**Background:**

Serogroups A/B/C/W/Y cause nearly all meningococcal disease. Therefore, comprehensive protection requires vaccination against all 5 serogroups. A pentavalent MenABCWY vaccine, composed of 2 licensed vaccines (MenB-fHbp, MenACWY-TT), is undergoing clinical development in adolescents and young adults. We predicted persistence of serum bactericidal activity against human complement (hSBA) titers 5 years after both a MenABCWY primary series and booster dose using a power law model (PLM).

**Methods:**

The PLM was fitted with hSBA data from a previous MenABCWY clinical trial (NCT03135834) assessing a 2-dose primary regimen (0, 6 months), immunopersistence 4 years post primary, and booster vaccination given observed titers in healthy 10–25-year-olds. The PLM was then used to predict hSBA titers against test strains up to 5 years after the primary series and up to 5 years after a booster dose. The hSBA lower limit of quantitation (LLOQ) was 1:16 for the A22 MenB strain and 1:8 for all other test strains.

**Results:**

The PLM-predicted hSBA titers were consistent with those observed after a 2-dose MenABCWY primary series and a booster dose, which was given 4 years after the primary series (**Figure 1–2**). At 5 years post primary series, the percentage of individuals with hSBA titers ≥ LLOQ was predicted to range from 63.4%‒100% for MenA/C/W/Y strains and 8.0%–26.8% for MenB strains. Five years after the booster dose, the PLM predicted that 97.0%–100% and 45.7%‒88.6% of individuals had MenA/C/W/Y and MenB hSBA titers ≥ LLOQ, respectively.

**Figure:**
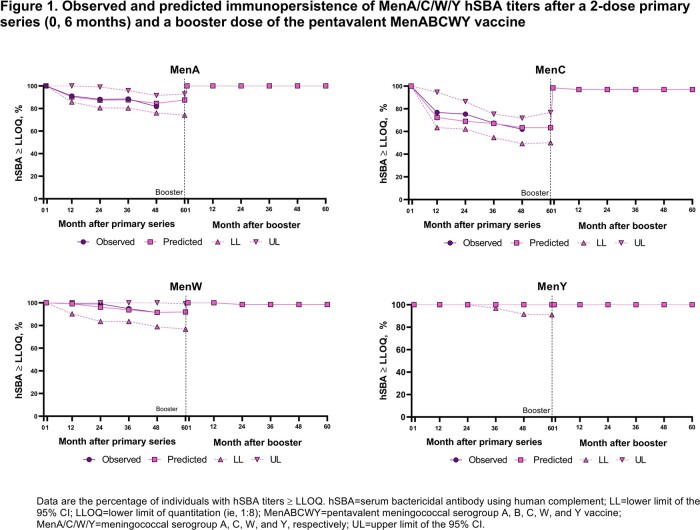

**Figure:**
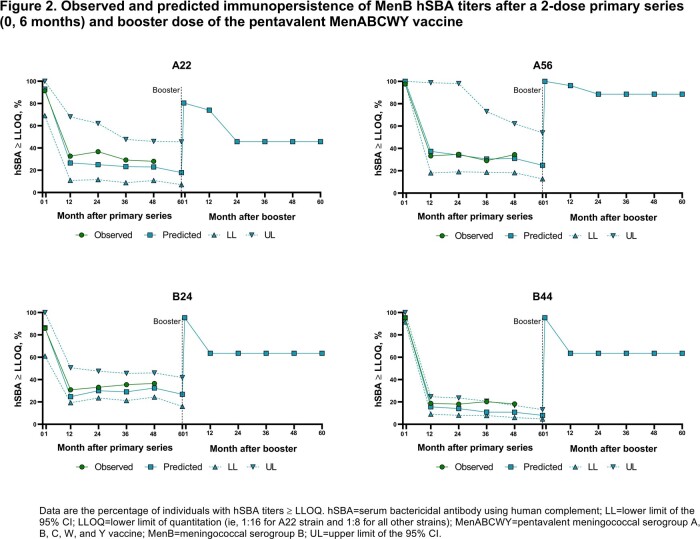

**Conclusion:**

The PLM supports that persistence of MenA/C/W/Y and MenB antibody responses after primary MenABCWY vaccination are maintained to at least 5 years. At 5 years after the booster dose, a substantial percentage of adolescents and young adults are predicted to have protective hSBA titers against all 5 serogroups.

Funding: Pfizer Inc

**Disclosures:**

**Bing Cai, PhD**, Pfizer Inc: Employee|Pfizer Inc: Stocks/Bonds **Paula Peyrani, MD**, Pfizer, Inc: Employee|Pfizer, Inc: Stocks/Bonds **Johannes Beeslaar, MD**, Pfizer Inc: Employee|Pfizer Inc: Stocks/Bonds **Jason Maguire, MD**, Pfizer, Inc.: Employee|Pfizer, Inc.: Stocks/Bonds **Roger Maansson, MS**, Pfizer Inc: Employee|Pfizer Inc: Stocks/Bonds **Paul Palmer, PhD**, Pfizer Inc: Employee|Pfizer Inc: Stocks/Bonds

